# Intracranial Subdural Hematoma Versus Postdural Puncture Headache Following Epidural Anesthesia: A Case Report

**DOI:** 10.7759/cureus.21824

**Published:** 2022-02-01

**Authors:** Dia R Halalmeh, Aubin Sandio, Munteanu Adrian, Marc D Moisi

**Affiliations:** 1 Neurosurgery, Hurley Medical Center, Flint, USA; 2 Internal Medicine, Detroit Medical Center, Wayne State University, Detroit, USA

**Keywords:** anesthesia, epidural, dural puncture, headache, subdural hematoma (sdh)

## Abstract

Headache is a relatively common complaint following dural puncture whether it is diagnostic (lumbar puncture) or unintentional (e.g., after epidural anesthesia). Although postdural puncture headache (PDPH) turns out to be the culprit in many cases, other serious etiologies should be ruled out such as postepidural intracranial subdural hematoma (PEISH). PEISH is usually overlooked because it is relatively rare and due to other frequent causes of headache (e.g., tension headache, migraine, and PDPH) being the main consideration. PEISH can be easily misdiagnosed as PDPH because of similar clinical manifestations. Herein, we report a case of this rare complication and demonstrate the major differences between PDPH and PEISH. This 27-year-old woman with intrauterine fetal death of dizygotic twins complained of severe headache immediately following receiving epidural anesthesia for labor induction. The patient was initially diagnosed with PDPH, and a blood patch was placed which provided complete resolution of the headache only for two days. Computed tomography of the brain revealed a small subdural hematoma over the left frontal convexity. Conservative management with close monitoring was recommended in this case due to the small size of the hematoma and absence of intracranial mass effect. An early follow-up CT scan showed complete and spontaneous resolution of the hematoma. In patients with recurrence or change in the pattern of the headache, persistence of headache despite treatment, and presence of neurological dysfunction following epidural anesthesia, suspicion of intracranial etiology must be raised. Therefore, knowledge of this condition and differentiating it from PDPH is necessary to avoid misdiagnosis and futile attempts of treatment.

## Introduction

Epidural block is one of the most frequent methods utilized in anesthesia. In general, it is a safe technique that can achieve adequate analgesia mainly in the lower body [1]. It is employed in a variety of procedures, most importantly, during childbirth in obstetric patients [1]. However, it is associated with several complications which can be very significant clinically such as intracranial subdural hematoma [2-11]. Many patients with subdural hematoma following epidural block can be mistakenly diagnosed with postdural puncture headache (PDPH). While intracranial subdural hematoma following epidural anesthesia is relatively rare (1:500,000) [2], it is critical for the physician to differentiate it from PDPH, as subdural hematoma can potentially lead to significant morbidity and mortality.

In this article, we report a case of small intracranial subdural hematoma following epidural block for labor induction of deceased dizygotic twins, describing the warning signs and symptoms and predisposing factors for this complication. This will allow for early diagnosis and intervention before neurologic compromise is established.

## Case presentation

A 27-year-old gravida 2 (G2) para 1 (P1) comes to the obstetric clinic for perinatal follow-up visit at 23 weeks’ gestation of monoamniotic-monochorionic twins. The patient reported decreased fetal movement earlier in the morning that day prior to the visit. Body surface ultrasound showed no cardiac motion for either fetus A or B and intrauterine fetal death (IUFD) likely due to cord entanglement. She was then admitted to Obstetrics and Gynecology department for induction of labor. The patient received epidural anesthesia and has since complained of postural headache and severe neck pain that radiated bilaterally to shoulders and midupper back. She stated that the headache is worsened by sitting upright and by moving her head to the right or to the left and improved when she lies down. She denies any fever, vomiting, confusion, or visual changes. Aside from neck pain, she complained of numbness in the right big toe, right lower extremity weakness, and lightheadedness. On physical examination, the patient was alert and oriented with no remarkable neurological signs except for mild weakness in the right lower extremity (4+/5). She was afebrile with normal body temperature, blood pressure, pulse, and respiratory rate (36.6°C (97.88°F), 106/79 mm Hg, 68/beat per minute (bpm), and 18/min, respectively).

**Figure 1:**
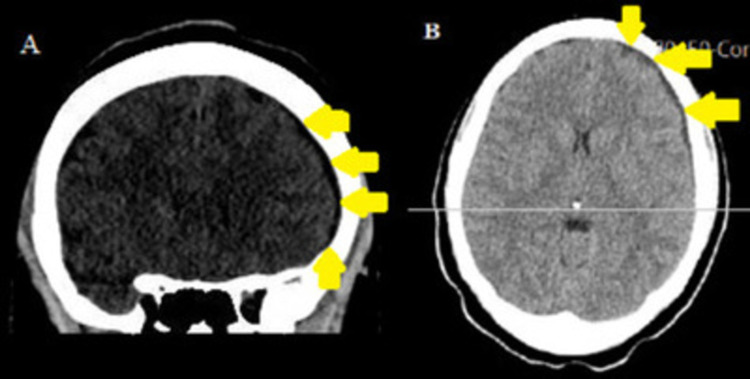
Left subdural hematoma Noncontrast coronal (A) and axial (B) CT scan of the brain demonstrating 4 mm thick subdural hematoma over the left frontal convexity (yellow arrows). This hematoma appears isodense to hypodense which is a characteristic of subacute subdural hematoma.

The patient was initially diagnosed with PDPH, and an epidural blood patch was then placed the next day. The headache resolved, but she was not able to move her neck due to pain. Nonsteroidal anti-inflammatory drugs (NSAIDs), spasmolytics, and caffeine pills were recommended which she claims helped alleviate the pain partially. An MRI of the cervical, thoracic, and lumbar spine was obtained and showed changes related to her recent blood patch; however, there was no significant canal stenosis. Brain CT scan with no contrast (Figures 1A, 1B) revealed left frontal convexity subdural hematoma measuring 4 mm in thickness with no midline shift, herniation, or associated mass effect.

Due to the relatively small size of the subdural hematoma and stability of the patient’s clinical condition, neurosurgical consultation considered conservative management. Two days after receiving the blood patch, the patient stated that the headache recurred with persistence of the pain and stiffness at the base of her neck. However, there was no clinical evidence of progressive neurological deterioration based on serial physical examinations. A repeat cranial CT scan was then obtained and demonstrated a stable appearance of the subdural hematoma with no signs of new intracranial hemorrhage or extra-axial fluid collection. Other etiologies and predisposing factors that may lead to subdural hematoma were ruled out. These include head injury, systemic diseases, bleeding disorders, and medications. The neck pain improved later with local heat and spasmolytic drugs, and headache resolved again with oral analgesics drugs. The patient was then discharged home on day seven with no significant neurological dysfunction.

**Figure 2:**
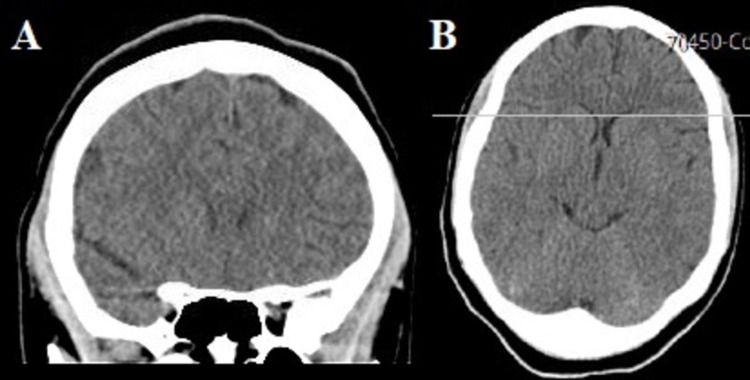
Radiological disappearance of subdural hematoma Noncontrast coronal (A) and axial (B) cranial CT scan showing complete resolution of the small left subdural hematoma.

One month later, the patient came for a neurology follow-up and reported that she is continuing to have headaches probably from her neck pain which is elicited upon turning her head around. She has been using pain killers to alleviate the pain with some relief. Other than the headache and neck pain, she denies any neurological symptoms or residual deficits from her previous episodes. During that evaluation, head CT scan was obtained and demonstrated spontaneous and complete resorption of the small subdural hematoma over the left frontal convexity. No abnormality was seen (Figures 2A, 2B).

## Discussion

Incidence and pathophysiology

The use of epidural anesthesia in obstetrical anesthesiology is well established. In general, complications associated with this kind of neuraxial anesthesia have been shown to be uncommon [12]. However, certain adverse events such as intracranial subdural hematoma are considered serious and can be potentially fatal if not recognized early and treated promptly. The incidence of postepidural intracranial subdural hematoma (PEISH) is unclear due to the retrospective nature of the data provided on these cases. However, an incidence of 1:500,000 has been estimated based on a large questionnaire involving 203 obstetric units in the United Kingdom [2]. Despite the great variation in reported incidence, acute subdural hematoma is not uncommon after dural puncture, especially in anticoagulated patients and patients with coagulation abnormalities. In such cases, a CT scan should be always obtained. Although both complications share similar clinical characteristics, PDPH is a more frequent sequela than PEISH. This may have contributed to the initial misdiagnosis of PDPH in our case. In a survey of 18,337 epidural blocks, 0.91% had accidental dural puncture with more than 88% of these developed PDPH [13]. After all, both PDPH and PEISH should be considered in any patient with postdural puncture headache.

In addition, PDPH and PEISH are very similar with respect to pathophysiology [2,4]. Cerebrospinal fluid (CSF) leakage typically occurs upon puncture of the dura mater. Excessive loss of the CSF leads to marked reduction in the CSF volume and subsequent intracranial hypotension [2,14]. This results in traction on the intracranial, pain-sensitive structures causing the headache. Stretching of the dural bridging veins (which are typically dilated to compensate for the CSF loss according to Monro-Kellie hypothesis) due to caudal displacement of the brain may lead to tear in the wall of subdural veins and consequently leaking blood in the subdural space with hematoma formation [2,3,6,14]. In our patient, the hematoma was most likely the result of unintentional dural puncture during the epidural block and probably continuous CSF leakage thereafter.

Clinical manifestation

As the name implies, the predominant symptom of PDPH is headache. The headache typically begins within the first three days (in about 90% of the patients) of the lumbar puncture [15]. Headache is generally severe, positional, aggravated by standing (postural) and movement of the head, relieved by lying in the supine position, and may radiate to shoulders and neck as seen in the current case. On the other hand, the headache associated with PEISH is usually nonpositional; however, a change in the nature of the headache has also been shown to be a clinical clue for intracranial etiology [3-6,14]. The presented case showed recurrence of the headache after disappearing which eventually appeared to be caused by intracranial subdural hematoma. Rapid neurological deterioration may be a characteristic of PEISH in severe cases [7]. Our patient was relatively stable with regard to neurological function likely due to the small hematoma (4 mm in thickness) and absence of compressive mass effect. Unlike the headache of PDPH which typically resolves within few days of conservative management (seven days in approximately 70% of patients [16]), PEISH headache is usually prolonged, severe, and resistant to conservative treatments.

Other nonspecific clinical findings of PDPH and PEISH include nausea, vomiting, visual and hearing disturbances, vertigo, and upper and lower extremity paresthesia. Although vomiting can be observed in both complications, it occurs more frequently with PEISH (31%-41% of cases) than with PDPH and is considered a warning sign of an intracranial complication in this case [2,4].

Risk factors

As both PDPH and PEISH share the same pathogenesis (puncture of the dura mater), both sequelae also share several common risk factors. These include female gender, history of similar previous episodes, history of chronic headache, obstetric patients like our patient who underwent labor induction for IUFD, size and shape of the needle (particularly large needles leading to excessive CSF leakage), low body mass index (BMI), dehydration, and experience of the operator [2,15]. There are numerous predisposing factors that specifically influence the development of intracranial subdural hematoma following induction of epidural anesthesia including coagulation abnormalities, anticoagulation agents, excessive alcohol consumption, and cerebral atrophy [2,8,9].

Diagnosis and management

Patients with severe persistent headache with or without focal neurological deficits after epidural anesthesia should be often suspected of having PEISH. The clinical diagnosis of PEISH is best confirmed by CT scan or MRI of the brain. Acute/subacute subdural hematomas such as PEISH classically appear as crescent-shaped, hyper-isodense, and extra-axial collection [2,5-7,17]. As MRI takes more time compared with CT scan, the latter is the preferred choice in the setting of emergent cases; however, MRI is reserved for inconclusive cases. Management is strongly dependent on the size of the hematoma and its resultant mass effect as well as the degree of impairment in the neurological function. Small hematomas do not typically produce a significant mass effect. As a consequence, conservative approach with close observation is usually sufficient to treat small collections, like in our patient, as the blood clot disintegrates and is reabsorbed normally with time. By contrast, PEISH with ≥1 cm in thickness, significant intracranial mass effect with midline shift or sulcal effacement, or rapid decompensation of neurological function requires immediate surgical evacuation [2,8]. This is often performed via either craniotomy or burr hole surgery.

The diagnosis of PDPH is based mainly upon the presence of suggestive clinical features, particularly postural headache, in a patient with either intentional or unintentional dural puncture [15]. Confirmation of the diagnosis is not usually necessary as this adverse event is typically self-limited within six weeks in about 85% of the cases [14]. However, we believe that a head CT scan should be performed where the clinical diagnosis is in doubt in order to exclude other serious etiologies that can mimic PDPH such as PEISH, as demonstrated in this article. Although conservative measures occasionally do not result in complete relief, conservative measures in the treatment of PDPH including bed rest, oral analgesics (e.g., NSAIDs, acetaminophen), caffeine pills, and rehydration help alleviate symptoms until resolved completely. Epidural blood patch has also been shown to be effective in the treatment of PDPH with excellent success rates and faster recovery [2,3,10]. On the contrary, efficacy of blood patching has been observed in the setting of PEISH only due to initial misdiagnosis and treatment as PDPH, prompting the placement of the blood patch. As seen in our patient, along with few reports in the literature [6,11,18], epidural blood patch resulted in disappearance of the associated headache for few days. The resolution of the headache after placement of epidural blood patch was likely due to blockage of further CSF leak. The major differences between PEISH and PDPH are summarized in Table 1.

**Table 1:** Comparison between PDPH and PEISH after epidural anesthesia PDPH: postdural puncture headache, PEISH: postepidural intracranial subdural hematoma, BMI: body mass index. *These may also contribute as predisposing factors for PEISH.

	PDPH	PEISH
Frequency	Very common.	Extremely rare.
Risk factors	Female gender, obstetric patients, large needles, history of previous episode, history of chronic headache, dehydration, and low BMI.*	Anticoagulants, coagulation abnormalities, cerebral atrophy, and alcohol abuse.
Clinical clues	Headache is positional, aggravated by standing upright, and alleviated by lying supine. Onset of the headache within the first few days after the lumbar puncture. Improvement of the headache within one week spontaneously without later recurrence. The use of epidural blood patch leads to faster recovery. No change in the pattern of the headache.	Headache is usually nonpositional, but postural headache can also occur. Persistence of the headache despite conservative treatment measures. Blood patching may relieve symptoms temporarily. Changes in the pattern of headache (e.g., change from postural to nonpostural, worsening of the headache). Episodes of headache that may disappear but recur later. Rapid neurologic deterioration.
Diagnosis	Typically made based on history and clinical evaluation.	Confirmed by CT scan along with presence of suggestive clinical features.
Management	Conservative approach (e.g., oral analgesics, caffeine pills, rehydration, bed rest). Epidural blood patch may be considered for those who failed the conservative management or want faster recovery.	Based on the size of the hematoma and stability of the clinical situation: If smaller than 10 mm, conservative treatment is recommended. If larger than 1 cm and/or there is neurological decompensation, surgical intervention is required.

## Conclusions

In summary, intracranial subdural hematoma consequent to epidural anesthesia is a clinically serious complication and is commonly misdiagnosed as PDPH. The index of suspicion for PEISH should be high, as this condition is life-threatening and can result in permanent neurological sequelae. Therefore, knowledge of the clinical features and clues of PEISH and early diagnosis are of paramount importance.
